# Definition of Occupational Lead Toxicity in Greece

**DOI:** 10.1289/ehp.10442

**Published:** 2007-10

**Authors:** Elias E. Mazokopakis, Theodore C. Constantinidis

**Affiliations:** Department of Internal Medicine, Naval Hospital of Crete, Chania, Crete, Greece, E-mail: emazokopakis@yahoo.gr; Laboratory of Hygiene and Environmental Protection, Medical School, Democritus University of Thrace, Alexandroupolis, Greece

We read with interest the article of Kosnett et al. (2007) in which the authors offered medical recommendations for the health management of lead-exposed adults. These recommendations were intended to apply to all workers who have the potential to be exposed by lead ingestion, even in the absence of documented elevations in air lead levels. Based on the literature and their experience, Kosnett et al. recommended that individuals be removed from occupational lead exposure if a single blood lead level (BLL) exceeded 30 μg/dL or if two successive BLLs measured over a 4-week interval were ≥ 20 μg/dL.

The definition of occupational lead toxicity is difficult because it is not possible to determine a precise BLL below which symptoms never occur, or a BLL at which symptoms are always reported. Moreover, individual susceptibility should always be recognized. Several attempts have been made over the years to define lead toxicity among adult workers based mainly on clinical investigations [Agency for Toxic Substances and Disease Registry (ATSDR) 2007].

In Greece the occupational exposure to lead is regulated by the Presidential Decrees 94/1987 1987 and 338/2001 2001. According to these decrees, the diagnosis of lead toxicity in adult workers is based on the integration of data obtained from the patient’s medical history, a physical examination, laboratory tests, and tests of specific organ function. An employee with a BLL of ≥ 40 μg/dL and exposure to lead at concentrations > 75 μg/m^3^ of air per 8-hr time-weighted average (TWA) require environmental and medical intervention (action levels). The maximum acceptable BLL for an adult worker is 70 μg/dL (BLL > 70 μg/dL should result in removal from lead exposure), but the permissible airborne exposure limit is < 150 μg/m^3^ per 8-hr TWA. *Presidential Decree 94/1987* 1987 prohibits “prophylactic” chelation for the prevention of elevated BLLs. Although these standards have provided guidance that has been beneficial for Greek occupational health physicians, they have not been substantially changed since 1987 [action level, BLL ≥ 50 μg/dL; maximum acceptable level, BLL = 70 μg/dL; permissible airborne exposure limit, air lead (workplace) < 150 μg/m^3^ (8-hr average)]. Therefore, a reformation of these standards is needed in Greece, on the basis of recent health effects studies, such as those on standards or regulations of health agencies.

Prevention of lead toxicity is a collaborative effort between primary care clinicians and public health agencies. Primary prevention is best achieved through the use of engineering controls, personal protective equipment, and good work practices. The occupational health physician can have the greatest impact on prevention through worker education and instruction in proper personal hygiene techniques. Also, the observance of up-to-date guidelines would contribute to the elimination of occupational lead toxicity in Greece.

## Editor’s note:

In accordance with journal policy, Kosnett et al. were asked whether they wanted to respond to this letter, but they chose not to do so.
